# Functional Neural Alterations in Pathological Internet Use: A Meta-Analysis of Neuroimaging Studies

**DOI:** 10.3389/fneur.2022.841514

**Published:** 2022-04-18

**Authors:** Wei Peng, Qinghong Hao, Heng Gao, Yang Wang, Jun Wang, Yang Tu, Siyi Yu, Hui Li, Tianmin Zhu

**Affiliations:** ^1^School of Acupuncture and Tuina, Chengdu University of Traditional Chinese Medicine, Chengdu, China; ^2^School of Rehabilitation and Health Preservation, Chengdu University of Traditional Chinese Medicine, Chengdu, China; ^3^Medical Quality Control Department, Chengdu Seventh People's Hospital, Chengdu, China; ^4^College of Traditional Chinese Medicine, Chongqing Medical University, Chongqing, China; ^5^School of Preclinical Medicine, Chengdu University, Chengdu, China

**Keywords:** pathological internet use, functional magnetic resonance imaging, SDM, meta-analysis, systematic review

## Abstract

Previous resting-state functional MRI (fMRI) studies found spontaneous neural activity in the brains of Pathological Internet Use (PIU) subjects. However, the findings were inconsistent in studies using different neuroimaging analyses. This meta-analytic study aimed to identify a common pattern of altered brain activity from different studies. Resting-state fMRI studies, based on whole-brain analysis methods published before July 1, 2021, were searched in multiple databases (PubMed, EMBASE, MEDLINE, and Web of Science). A voxel-based signed differential mapping (SDM) method was used to clarify brain regions, which showed anomalous activity in PIU subjects compared with healthy controls (HCs). Ten eligible publications consisting of 306 PIU subjects and 314 HCs were included in the SDM meta-analysis. Compared with HCs, subjects with PIU showed increased spontaneous neural functional activity in the left temporal pole of the superior temporal cortex, left amygdala, bilateral median cingulate cortex, and right insula. Meanwhile, a decreased spontaneous neural activity was identified in the left dorsolateral superior frontal gyrus and right middle frontal gyrus in the subjects with PIU. These abnormal brain regions are associated with cognitive executive control and emotional regulation. The consistent changes under different functional brain imaging indicators found in our study may provide important targets for the future diagnosis and intervention of PIU.

**Systematic Review Registration:** www.crd.york.ac.uk/PROSPERO, identifier: CRD42021258119.

## Introduction

As related industries, such as smart devices, mature, the Internet has become an essential tool for learning, working, and playing. According to Internet World Stats (IWS) (1), as of March 2021, the number of Internet users has reached 5.17 billion, with Asia accounting for 53.4% of the world's Internet users. Such a high Internet penetration rate has brought a severe social problem, namely Pathological Internet Use (PIU). The PIU refers to an inability to control one's Internet use that adversely affects daily life, also known as “Internet Addiction (IA)” (2–4). The global average prevalence is about 7.02% and is still on the rise (5). Previous studies have suggested that PIU has a similar neuropathological basis to substance addiction (6, 7). Although more attention has been paid to PIU, the pathological mechanism of PIU is still unclear.

Functional MRI (fMRI) is a common neuroimaging technique to explore the neuropathological mechanism of diseases (8). Some fMRI indicators based on whole-brain analysis, such as functional connectivity (FC), the amplitude of low-frequency fluctuation (ALFF), regional homogeneity (ReHo), functional connection density (FCD), voxel-mirrored homotopic connectivity (VMHC), and cerebral blood flow (CBF), provided technical support for the comprehensive exploration of spontaneous neural activity in the brain of subjects with PIU. Previous studies have suggested that the limbic system, which is involved in the reward-processing circuit, and the prefrontal lobe, which is involved in the cognitive control circuit, are the physiological basis for the formation of PIU (9–12). However, these findings have been controversial in resting-state fMRI studies. For example, some researchers found increased spontaneous brain activity in the superior frontal gyrus (SFG) of subjects with PIU (13), while others found a decreased neural activity in this region (14, 15). In addition, the neural activity in superior temporal gyrus (STG) of subjects with PIU in resting-state was found to be increased (13), while another research found it decreased (16). The inconsistent results might be related to the differences in sample size, imaging analysis indexes, and demographic characteristics. These differences make it difficult to understand the neural mechanism of PIU, so further quantitative exploration is needed.

A systematic and quantitative meta-analysis, such as signed differential mapping (SDM) analysis, can find consistent local resting-state abnormalities regardless of all method differences (17). The SDM meta-analysis method could address heterogeneity between studies by reconstructing positive and negative graphs in the same image, thereby counterbalancing the effect of reporting findings in opposite directions (18). This voxel-based neuroimaging meta-analysis method has been validated in psychiatric disorders, such as depression, autism, and behavioral addiction (19–21). Moreover, researchers have used this method to find consistent activation results of brain regions in subjects with PIU in task-state fMRI (22–24). However, the changes of spontaneous brain activity of PIU in the resting state remain to be explored.

Hence, in this study, we aimed to unearth the consistency of changes in spontaneous brain functional activity in subjects with PIU during resting state. The findings of this meta-analysis will help to understand the pathological basis of PIU and give evidence for PIU prevention and intervention in the future.

## Materials and Methods

This study was reported according to preferred reporting items for systematic review and meta-analysis (PRISMA) guidelines (25) and registered on PROSPERO (registration No: CRD42021258119).

### Search Strategy

We searched PubMed, EMBASE, MEDLINE, and Web of Science (WOS) databases for publications published before July 1, 2021. The following search terms and their derivatives were used: (“pathological Internet use” OR “problematic Internet use” OR “Internet addiction” OR “Internet addiction disorder” OR “Internet use” OR “gaming addiction” OR “Internet gaming disorder” OR “mobile phone addiction” OR “smartphone addiction” OR “Internet dependence” OR “mobile phone dependence” OR “smartphone dependence”) AND (“magnetic resonance imaging” OR “MRI” OR “functional magnetic resonance imaging” OR “fMRI”). In addition, we manually searched the list of references included in the study for other possible articles. The study was not restricted by country, year of publication, or publication status.

### Inclusion and Exclusion Criteria

Studies were included according to the following eligibility criteria: (i) participants were diagnosed with any of the accepted diagnostic criteria for PIU and were not limited by age, sex, or race; (ii) using resting-state fMRI technique; (iii) peer-reviewed; (iv) studies that reported standard three-dimensional spatial coordinates, such as Talairach/Tournoux Spaces or Montreal Neurological Institute (MNI) Spaces; (iv) fMRI studies using whole-brain analysis, including whole-brain FC, ALFF, ReHo, CBF, independent component analysis (ICA), degree centrality (DC), etc.; (v) original, cross-sectional comparative studies (subjects with PIU compared with healthy people). The following types of studies were excluded: (i) participants with other types of addictive or psychiatric disorders; (ii) literature lacking anatomical coordinates for the main results; (iii) repeated publications; (iv) studies with a sample size <15 cases.

### Data Extraction

According to the literature retrieval method, the researchers (JW and QHH) independently downloaded the literature that met the requirements and removed the duplicates through Endnote software. After careful reading of the abstract and full text, studies that met the inclusion criteria were screened out. Any differences were resolved through discussion by the third (WP) researcher until a consensus was reached. After that, the Microsoft Excel spreadsheets were built to extract data from the articles. For any missing data in the article, we requested the original authors *via* email if necessary. If the study was a longitudinal study design, only baseline data were included in our analysis. Additionally, for the studies published from the same team using the same batch of data, only the latest published studies or studies with a large sample size were included.

The data we extracted from each study were as follows: (i) the characteristics of the study: first author, year of publication, and country; (ii) the characteristics of participants: sample size, age, sex, inclusion criteria, diagnostic methods used, and the severity of PIU; (iii) neuroimaging methods and results: scanner strength and brand, head coil (number of channels), the fMRI data analysis method, peak coordinates of activated brain regions, etc.

### Assessment of Methodological Quality

The quality of all included studies was assessed by the Newcastle-Ottawa scale (NOS) (26). The NOS includes two types of quality evaluation lists: the case-control studies and cohort studies. Each scale contains three dimensions (selection, comparability, and outcome) and a total of 8 questions. The scale's overall score ranges from 0 to 9, with studies scoring seven or more are considered high-quality (27).

### Meta-Analysis

Before the SDM meta-analysis, we will conduct a descriptive analysis of all the included studies. Then, a voxel-based meta-analysis was performed on the included literature using SDM software (http://www.sdmproject.com) to evaluate the differences in brain activity between participants with PIU and healthy controls. Firstly, according to the requirements of the SDM software manual (www.sdmproject.com/manual/), the sample size, peak coordinates, corresponding *t* value of peak points, and threshold information of each study were imported into the software. For studies where statistical *t-*values were not reported, SDM provided a converter to convert statistical values such as *z* or *p-*values to *t* values. Secondly, for each study, standard Talairach maps (with both positive and negative effect sizes) of gray matter differences were recreated separately. Thirdly, random effect analysis was performed to obtain the mean maps of all studies (28). In our study, the statistically significant threshold was set to at least 20 voxels and uncorrected *p* < 0.005. Radua et al. (29) suggested that the sensitivity and specificity of the study results could maintain the optimal balance when the *P*-value was set at 0.005. This threshold has also been applied in previous meta-analysis studies (30, 31).

The heterogeneity among the original studies was tested using the random-effects model of *I*^2^ statistics. When *I*^2^ statistics were 0, 25, 50, and 75%, it represented non-heterogeneity, low heterogeneity, medium heterogeneity, and high heterogeneity, respectively. Besides, meta-regression analysis was used to understand the source of heterogeneity between studies. The threshold of meta-regression analysis was set as *p* < 0.0005 and cluster size ≥ 10 voxels (28). Additionally, to verify the stability of the results of the meta-analysis, the leave-one-out jack-knife sensitivity analysis will be used. Finally, MRIcroGL software was used to present brain clusters with significant differences in the MNI standard spatial template.

## Results

**Figure 1:**
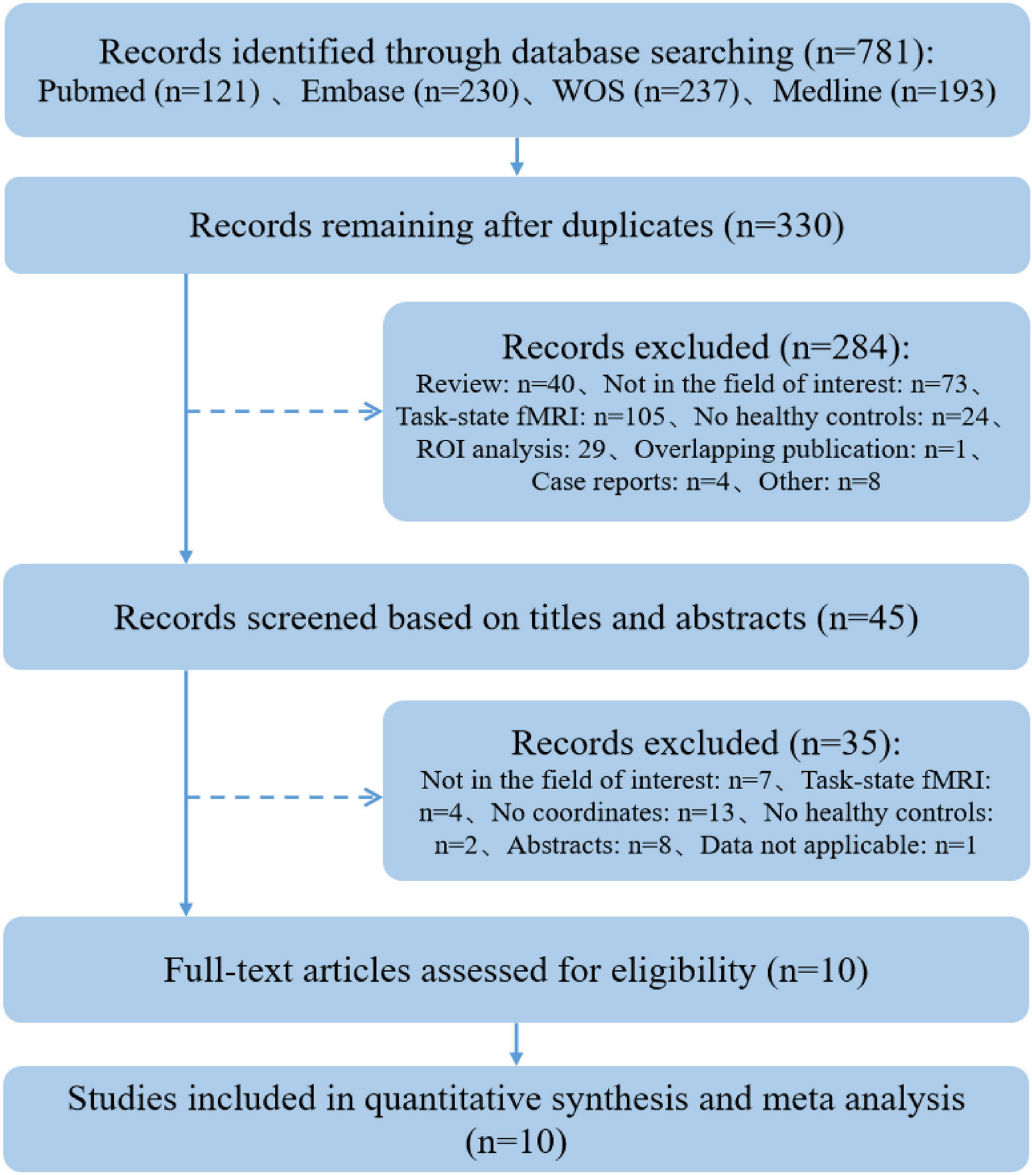
A flow chart of the study selection process.

A total of 781 articles were retrieved. After the screening procedure, 10 studies (13–16, 32–37) met eligibility criteria and were included in the systematic review (see Figure 1).

### Description of the Included Studies

About 306 subjects with PIU (252 males) and 314 healthy controls (HCs) (251 males) were included in the systematic review (see Table 1 for study details). The subjects with PIU were well-matched in age and gender distribution with healthy controls. All of the studies involved people under the age of 30, and five included adolescents (15, 32–34, 36). Studies included more male participants than female participants, and four included only male participants (16, 32, 34, 35). Of the ten studies, nine were from China (13–15, 32–37) and one from South Korea (16). Ten studies comprised the following groups with PIU: internet gaming disorder (IGD) groups (*n* = 6) (14–16, 32, 34, 35), the internet gaming addiction (IGA) group (*n* = 1) (33), the IA group (*n* = 2) (36, 37), and the internet addiction disorder (IAD) group (*n* = 1) (13). Three studies used Young's Internet Addiction Test (IAT) (16, 32, 37), and five studies used the Chen Internet Addiction Scale (CIAS) (14, 15, 33–35) to evaluate the severity of Internet addiction.

**Table 1:** Demographics and clinical characteristics included in this study.

**First author (publication year)**	**Country**	**Types of PIU**	**Sample size**		**Gender (M/F)**		**Age (years)**		**Clinical characteristics of PIU**	
			**PIU**	**HCs**	**PIU**	**HCs**	**PIU**	**HCs**	**IAT Score**	**CIAS Score**
Xin Du (32)	China	IGD	27	35	27/0	35/0	17.07 ± 3.55	16.80 ± 2.34	68.19 ± 11.79	–
Qi Feng (33)	China	IGA	15	18	13/2	14/4	16.93 ± 2.34	16.33 ± 2.61	–	66.73 ± 3.01
Xu Han (34)	China	IGD	26	30	26/0	30/0	16.81 ± 0.75	17.00 ± 0.89	–	71.88 ± 5.56
Heejung Kim (16)	Korea	IGD	16	15	16/0	15/0	21.63 ± 5.92	25.40 ± 5.29	75.81 ± 4.72	–
Jun Liu (13)	China	IAD	19	19	11/8	11/8	21.00 ± 1.30	20.00 ± 1.80	–	–
Lu Liu (35)	China	IGD	74	41	74/0	41/0	22.28 ± 1.98	23.02 ± 2.09	–	78.46 ± 8.40
Yawen Sun (14)	China	IGD	53	52	30/23	30/22	21.87 ± 3.08 (M)	20.73 ± 2.16 (M)	–	74.43 ± 9.19 (M)
							21.91 ± 2.92 (F)	21.09 ± 3.85 (F)		74.35 ± 9.21 (F)
Yao Wang (15)	China	IGD	17	24	13/4	18/6	16.94 ± 2.73	15.87 ± 2.69	–	64.59 ± 6.43
Lubin Wang (36)	China	IA	31	50	21/10	35/15	15.00 ± 1.30	15.10 ± 0.50	–	–
Yang Wang (37)	China	IA	28	30	21/7	22/8	21.32 ± 1.96	21.73 ± 2.08	73.89 ± 6.76	–

*F, female; M, male; HCs, healthy controls; PIU, pathological Internet use; CIAS, Chen Internet addiction scale; IAT, young's Internet addiction test; IGD, Internet gaming disorder; IGA, internet gaming addiction; IA, internet addiction; IAD, internet addiction disorder*.

Table 2 provides detailed information on the research methods used in each study. Six studies (13, 14, 32, 34, 36, 37) used one diagnostic method for PIU, and three studies (15, 16, 33) used two diagnostic methods for PIU. Notably, among the diagnostic criteria they used, the modified Young's Diagnostic Questionnaire for Internet Addiction criteria by Beard (38) was the most widely used. All studies used a 3T MRI scanner to acquire data. There were two studies for whole-brain FC analysis (15, 35), two studies for ReHo analysis (13, 16), two studies for ALFF analysis (14, 34), two studies for FCD analysis (32, 37), one study for ICA (36), and one study for CBF analysis (33).

**Table 2:** Methodological characteristics included in the study.

**First author (year)**	**Diagnostic criteria**	**MRI scanner**	**Methods**	**MRI head coil**	**Research types**	**NOS quality score**
Xin Du (32)	Young's Diagnostic Questionnaire for IA	Siemens MRI scanner (3T)	FCD	–	Case-control	5
Qi Feng (33)	(1) DSM-IV (2) The modified Diagnostic Questionnaire for IA criteria by Beard	GE MRI scanner (3T)	CBF	Standard	Case-control	6
Xu Han (34)	The modified Diagnostic Questionnaire for IA criteria by Beard	GE MRI scanner (3T)	ALFF, seed-based FC	Standard	Case-control	5
Heejung Kim (16)	(1) DSM-V (2) YIAT	Philips MRI scanner (3T)	ReHo	Standard	Case-control	7
Jun Liu (13)	The modified Diagnostic Questionnaire for IA criteria by Beard	Siemens MRI scanner (3T)	ReHo	Standard	Case-control	5
Lu Liu (35)	–	Siemens MRI scanner (3T)	FC	–	Case-control	5
Yawen Sun (14)	The modified Diagnostic Questionnaire for IA criteria by Beard	GE MRI scanner (3T)	ALFF, seed-based FC	Standard	Case-control	6
Yao Wang (15)	(1) DSM-IV (2) The modified Diagnostic Questionnaire for Internet Addiction criteria by Beard	GE MRI scanner (3T)	FC	Standard	Case-control	6
Lubin Wang (36)	The modified Diagnostic Questionnaire for IA criteria by Beard	Philips MRI scanner (3T)	ICA	–	Case-control	8
Yang Wang (37)	Young's Diagnostic Questionnaire for IA	GE MRI scanner (3T)	FCD	Standard	Case-control	6

*ALFF, amplitude of low-frequency fluctuation; CBF, cerebral blood flow; DSM, the diagnostic and statistical manual of mental disorders; FC, functional connectivity; FCD, functional connectivity density; IGD, Internet gaming disorder; IA, internet addiction; IAD, internet addiction disorder; ICA, independent component analysis; IGA, internet gaming addiction; NOS, Newcastle-Ottawa scale; PIU, pathological Internet use; ReHo, regional homogeneity; YIAT, young's Internet addiction test*.

### Quality Assessment Results

The average NOS score for the ten studies was 6.1 (see Table 2 and Supplementary Table S1). Two case-control studies (16, 36) were considered high quality, with NOS scores above 7. Among the remaining case-control studies, six had NOS scores of 6 (14, 15, 33–35, 37), and two had NOS scores of 5 (13, 32).

### Main Meta-Analysis Results

**Figure 2:**
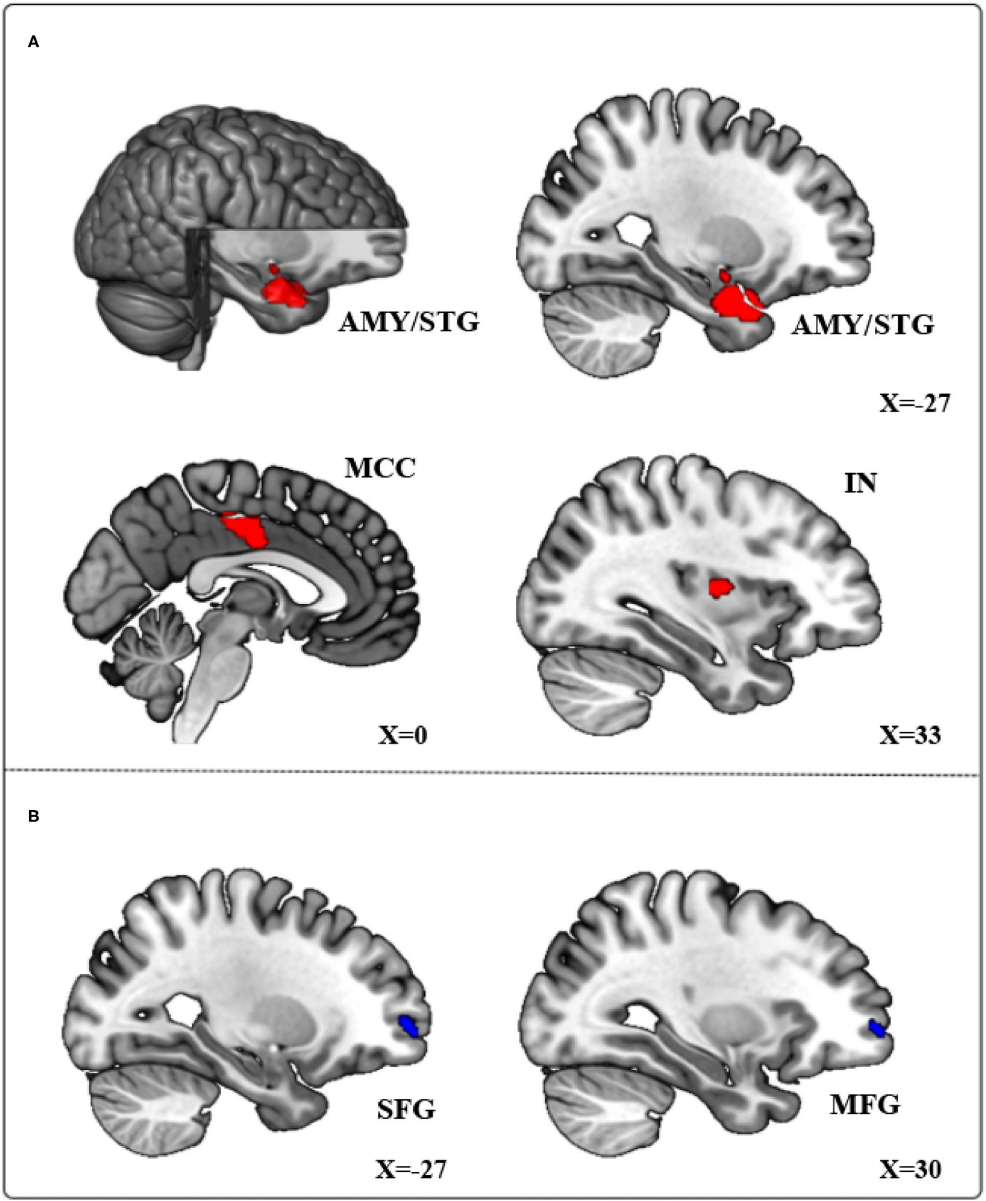
Meta-analytical results of the contrast of Pathological Internet Use (PIU) vs. healthy controls (HC). **(A)** Red regions showing significant increases in the left temporal pole of the STG (STGtp), left amygdala (AMY), bilateral median cingulate cortex (MCC), and right insula (IN). **(B)** Blue regions showing significant decreases in the left dorsolateral SFG (SFGdl) and right middle frontal gyrus (MFG).

The primary meta-analysis results were summarized in Table 3 and Figure 2. Compared with HCs, subjects with PIU had increased spontaneous neural activity in the left temporal pole of the STG (STGtp)/left amygdala (AMY) (408 voxels, peak coordinate: −26, 4, −26), bilateral median cingulate cortex (MCC) (364 voxels, peak coordinate: 0, −12, 38), and right insula (IN) (21 voxels, peak coordinate: 34, −6, 10). Decreased spontaneous neural activity in subjects with PIU was seen in the left dorsolateral SFG (SFGdl) (30 voxels, peak coordinate: −28, 60, 0) and right middle frontal gyrus (MFG) (20 voxels, peak coordinate: 32, 58, 0).

**Table 3:** Abnormal resting state neural activity in subjects with Pathological Internet Use (PIU) compared with healthy controls (HC) (voxels ≥ 20).

**Cluster No**.	**Voxels (voxels)**	**SDM-Z**	***P*-value**	**Brain region**	**Brodmann area**	**MNI coordinate**			***I* ^2^**	**Meta bias test (*p*-value)**
						**x**	**y**	**z**		
PIU > HCs
1	408	3.820	0.00007	(Undefined)	BA 28	−26	4	−26	7.20%	0.922
		3.705	0.00011	LSTGtp	BA 38	−26	12	−30		
		3.531	0.00021	L AMY	BA 28	−22	−4	−24		
		2.953	0.00158	L AMY	BA 34	−30	−4	−14		
		2.823	0.00238	L AMY	BA 34	−26	−2	−14		
2	364	3.609	0.00015	L MCC	BA 23	0	−12	38	8.04%	0.683
		3.420	0.00031	L MCC	BA 23	−4	−22	44		
		3.388	0.00035	R MCC	BA 23	2	−10	34		
		3.371	0.00037	R MCC	BA 23	8	−20	42		
3	21	2.953	0.00157	R IN	BA 48	34	−6	10	2.15%	0.999
PIU < HCs
4	30	−3.110	0.00093	L SFGdl	BA 11	−28	60	0	23.33%	0.489
5	20	−3.199	0.00068	R MFG	BA 11	32	58	0	17.21%	0.5

*L, left; R, right; HCs, healthy controls; PIU, pathological Internet use; AMY, Amygdala; BA, Brodmann area; IN, insula; MCC, median cingulate cortex; MFG, middle frontal gyrus; MNI, Montreal neurological institute; SFGdl, dorsolateral superior frontal gyrus; STGtp, temporal pole of the superior temporal gyrus; 1 voxel was 2mm × 2mm × 2mm*.

After SDM meta-analysis, we conducted heterogeneity analysis and meta-bias test for the statistically significant brain regions above. As shown in Table 3, the *I*^2^ statistics of all peak coordinate brain regions were <25%, indicating a low heterogeneity. In addition, meta-bias test results showed no publication bias in each peak voxel level (*p* > 0.05). After meta-regression analysis, we found no significant influence of age and gender on the main findings. Due to the different types of other clinical variables reported in each study (such as time spent online, medication, etc.), these clinical variables were not included in the meta-regression analysis.

### Sensitivity Analysis Results

Jack-knife sensitivity analysis was to remove one study, in turn, and re-conduct mean analysis on the remaining studies to judge the stability of SDM meta-analysis results. A total of 10 mean analyses were performed in this systematic review. As shown in Table 4, the cluster results of the four brain regions (left STGtp/left AMY, left MCC, left SFGdl, right MFG) showed high stability.

**Table 4:** Results of sensitivity analysis of Jack-Knife.

**First author (year)**	**Increased brain regions**			**Decreased brain regions**	
	**L STGtp/L AMY**	**L MCC**	**R IN**	**L SFGdl**	**R MFG**
Xin Du (32)	√	√	×	√	√
Qi Feng (33)	√	√	×	√	√
Xu Han (34)	√	√	×	√	√
Heejung Kim (16)	√	×	×	√	√
Jun Liu (13)	√	√	√	√	√
Lu Liu (35)	√	√	√	√	√
Yawen Sun (14)	√	√	√	×	×
Yao Wang (15)	√	√	√	×	×
Lubin Wang (36)	√	√	√	√	√
Yang Wang (37)	×	×	√	√	√

*√, the brain region was still present after the study was removed; ×, the brain region was not included in the results after the study was removed; L, left; R, right; AMY, Amygdala; IN, insula; MCC, median cingulate cortex; MFG, middle frontal gyrus; SFGdl, dorsolateral superior frontal gyrus; STGtp, temporal pole of the superior temporal gyrus*.

## Discussion

This study is the first to use quantitative SDM meta-analysis to integrate abnormal neural activity in resting-state fMRI studies of subjects with PIU. We found that local neural activity of STGtp, AMY, MCC, and IN increased in the subjects with PIU, while the local neural activity of SFGdl and MFG decreased. These abnormal brain regions were closely related to the cognitive executive control and emotional regulation functions of the subjects with PIU. Our findings provided a reference for exploring the pathological mechanism of PIU.

Executive control dysfunction is a common feature in the subjects with PIU (39). In our study, multiple brain regions are involved in the executive control function of the brain, mainly including STGtp, SFGdl, and MFG. The STGtp is an important component of the temporal lobe, located in front of STG, and participates in a variety of important cognitive functions (40). Studies have found that the patients with cognitive impairment had reduced STG cortical thickness and increased neural activity compared with normal subjects (41–43). Besides, a recent neuroimaging meta-analysis of PIU has shown that STG was hyperactivated in the executive control tasks, suggesting that STG was involved in the executive control function of the subjects with PIU (24). These studies suggested that increased neural activity of STGtp in the subjects with PIU in the resting state may be a compensatory mechanism for executive control function deficits in the subjects with PIU.

The SFGdl and MFG were also important to brain regions for the executive control function. These regions were located in the frontal cortex, associated with the executive control network, and regulated other cognitive functions (44–46). A previous meta-analysis found that the subjects with PIU showed abnormal frontal cortex activation in multiple cognitive tasks (22). A recent study has revealed that the reduced SFG gray matter volume played a mediating role in the influence of emotional reflection in PIU (47). Other studies found that abnormal activation of SFG and MFG might be involved in the control of impulsivity in the subjects with PIU (48, 49). In our study, the decreased neural activity in SFGdl and MFG in the subjects with PIU may indicate the decreased control ability of the subjects with PIU to impulsivity.

Mood changes are another common complication in the subjects with PIU (50). Our meta-analysis showed that there were brain regions associated with emotion regulation, such as the AMY. This finding is consistent with previous reports of AMY dysfunction in PIU (51, 52), as well as with other addictions (53, 54). The AMY is an important component of the basal ganglia and is involved in integrating and processing information about emotions and rewards (55–57). Previous studies have suggested that AMY could regulate both positive and negative emotions (58). Subsequent studies have found the role of the central nucleus of the AMY in negative emotions accompanying reward loss (59) and the effect of the glutamate pathway from basolateral AMY to nucleus accumbens (NAc) on controlling reward-seeking behaviors (60). Therefore, the increase in spontaneous neural activity in AMY reflects the higher susceptibility to mood changes in the subjects with PIU and abnormalities in the reward system in the brain of the subjects with PIU.

Additionally, abnormalities in the salience network (e.g., IN, MCC) were found in the subjects with PIU. The salience network is a neural system for perceiving and responding to homeostatic demands and is closely related to diseases such as addiction and depression (61–63). The IN and cingulate are the key nodes of the Salience network (64). Previous studies have found that subjects with PIU have increased cortical thickness in IN (65), while decreased gray matter density in IN and MCC than HCs (66–68). These findings suggest structural abnormalities in the salience network of the subjects with PIU, which may lead to functional problems of the salience network. A recent meta-analysis of behavioral addiction has also found that neural activity in MCC increased when behavioral addicts were exposed to addiction-related cues (69). Furthermore, neural activity in the IN significantly increased when the subjects with PIU were exposed to game cues (70, 71). Therefore, the salience network abnormalities we found in the subjects with PIU were related to their excessive craving for addictive cues.

Furthermore, our study confirmed that PIU and addiction had similar neuronal activation patterns. The results of a systematic review of addiction showed that the changes of spontaneous neural activity in frontal and temporal regions were found in both substance addiction and behavior addiction (72, 73). These abnormal functional activity changes in brain areas were mainly related to emotion and cognitive control. This suggested that both PIU and addiction showed functional impairments in brain regions associated with cognitive and emotional processing. The difference was that the patients with substance addiction showed spontaneous changes in neural activity in the striatum in their resting state (72). Although the striatum was also found to be involved in the processing of rewards in the subjects with PIU during task fMRI studies (74, 75). However, in our study, no consistent neural activity was found in the striatum of the subjects with PIU in the resting state. Moreover, these findings were indirect rather than direct comparisons of neuronal activity patterns between PIU and addiction. More research is needed in the future to explore differences in neuronal activity patterns between PIU and other addictive disorders.

There are several limitations to this study. The first is that our study only included fMRI data from the whole-brain analysis, not data based on the region of interest (ROI) analysis. As different researchers choose different ROIs, this also increases the difficulty of data combination. Thus, whole-brain analysis studies avoid inconsistencies in the choices of ROI to researchers. Second, the studies we included used different analysis methods and imaging modalities. Hence, the heterogeneity due to method differences cannot be completely excluded. For example, the results of FC, FCD, and ICA analysis can reflect the connections between different brain regions or brain networks. The results of the ReHo and ALFF methods can reflect the characteristics of local neural activity in the brain (76, 77). The CBF is the main indicator of arterial spin labeling (ASL). The ASL is also an fMRI technique, which can reflect the brain metabolism and neural activity of subjects in the resting state (78). However, the research results of different modes and analytical methods could reflect the situation of a certain field more comprehensively. In addition, previous fMRI meta-analysis studies also combined fMRI studies with different analysis methods (79, 80). Therefore, when the number of studies using a single analysis method is not enough, it is feasible to combine the results of fMRI studies using multiple analysis methods. Second, due to the small number of included studies, the results might be subject to random error. It is worth noting that the heterogeneity test showed low heterogeneity in the results of this study. Sensitivity analysis also suggested that the results of this study were highly reproducible. Therefore, our study could be used as a preliminary study to reflect the characteristics of spontaneous neural activity changes in the brain of the subjects with PIU in resting state. Third, PIU may have some comorbidities (such as anxiety and depression disorders) that were not considered in our study. Due to the small number of studies that we included, a subgroup analysis was not possible. Finally, the cases in our study were all Asian. Therefore, our results can only represent the neuroimaging characteristics of the subjects with PIU in Asia. We also look forward to further neuroimaging studies of PIU in more countries.

## Conclusion

Our study identified consistent changes in brain regions in subjects with PIU from different fMRI studies. The subjects with PIU showed abnormal functional activity in brain regions and functional brain networks involved in cognitive executive control and emotional regulation, which constitute the core symptoms of PIU. These consistent changes in brain regions may provide important targets for the future diagnosis and intervention of PIU.

## Data Availability Statement

The original contributions presented in the study are included in the article/Supplementary Material, further inquiries can be directed to the corresponding author/s.

## Author Contributions

WP, SY, HL, and TZ designed this study. WP, QH, HG, JW, and YT were involved in the process of literature selection, data collection, and quality assessment. WP performed the data analysis and wrote the manuscript. SY, YW, HL, and TZ critically revised the work. All authors have approved the final manuscript.

## Funding

This work was supported by the National Natural Science Foundation of China (81072852 and 81574047), the Key R&D Project of Sichuan Province (2019YFS0175), the Xinglin Scholars Scientific Research Promotion Program of Chengdu University of Traditional Chinese Medicine (XSGG2019007), and the Training Funds of Academic and Technical Leader in Sichuan Province.

## Conflict of Interest

The authors declare that the research was conducted in the absence of any commercial or financial relationships that could be construed as a potential conflict of interest.

## Publisher's Note

All claims expressed in this article are solely those of the authors and do not necessarily represent those of their affiliated organizations, or those of the publisher, the editors and the reviewers. Any product that may be evaluated in this article, or claim that may be made by its manufacturer, is not guaranteed or endorsed by the publisher.

## References

[1] Internet World Stats. World Internet Users and 2021 Population Stats. (2021). Available online at: https://internetworldstats.com/stats.htm (accessed March 31, 2021).

[2] Young KS. Internet addiction - A new clinical phenomenon and its consequences. Am. Behav. Scientist. (2004) 48:402–15. 10.1177/0002764204270278

[3] Li W O'Brien JE Snyder SM Howard MO. Characteristics of internet addiction/pathological internet use in U.S. university students: a qualitative-method investigation. PLoS ONE. (2015) 10:e0117372. 10.1371/journal.pone.0117372 25647224 PMC4315426

[4] Greenfield DN. Psychological characteristics of compulsive internet use: a preliminary analysis. Cyberpsychol Behav. (1999) 2:403–12. 10.1089/cpb.1999.2.403 19178212

[5] Pan YC Chiu YC Lin YH. Systematic review and meta-analysis of epidemiology of internet addiction. Neurosci Biobehav Rev. (2020) 118:612–22. 10.1016/j.neubiorev.2020.08.013 32853626

[6] Sun YK Sun Y Lin X Lu L Shi J. Similarities and differences in neuroimaging. Adv Exp Med Biol. (2017) 1010:73–89. 10.1007/978-981-10-5562-1_5 29098669

[7] Zou Z Wang H d'Oleire Uquillas F Wang X Ding J Chen H. Definition of substance and non-substance addiction. Adv Exp Med Biol. (2017) 1010:21–41. 10.1007/978-981-10-5562-1_2 29098666

[8] Rosen BR Savoy RL. fMRI at 20: has it changed the world? Neuroimage. (2012) 62:1316–24. 10.1016/j.neuroimage.2012.03.004 22433659

[9] Wang L Yang G Zheng Y Li Z Qi Y Li Q . Enhanced neural responses in specific phases of reward processing in individuals with Internet gaming disorder. J. Behav. Addict. (2021) 10:99–111. 10.1556/2006.2021.00003 33570505 PMC8969865

[10] Yao YW Liu L Worhunsky PD Lichenstein S Ma SS Zhu L . Is monetary reward processing altered in drug-naive youth with a behavioral addiction? Findings from internet gaming disorder. Neuroimage Clin. (2020) 26:102202. 10.1016/j.nicl.2020.102202 32045732 PMC7013339

[11] Sepede G Tavino M Santacroce R Fiori F Salerno RM Di Giannantonio M. Functional magnetic resonance imaging of internet addiction in young adults. World J Radiol. (2016) 8:210–25. 10.4329/wjr.v8.i2.210 26981230 PMC4770183

[12] Li Q Wang Y Yang Z Dai W Zheng Y Sun Y . Dysfunctional cognitive control and reward processing in adolescents with Internet gaming disorder. Psychophysiology. (2020) 57:e13469. 10.1111/psyp.13469 31456249

[13] Liu J Gao XP Osunde I Li X Zhou SK Zheng HR . Increased regional homogeneity in internet addiction disorder: a resting state functional magnetic resonance imaging study. Chin Med J. (2010) 123:1904–8. 10.3760/cma.j.issn.0366-6999.2010.14.014 20819576

[14] Sun Y Wang Y Han X Jiang W Ding W Cao M . Sex differences in resting-state cerebral activity alterations in internet gaming disorder. Brain Imaging Behav. (2019) 13:1406–17. 10.1007/s11682-018-9955-4 30178423

[15] Wang Y Yin Y Sun YW Zhou Y Chen X Ding WN . Decreased prefrontal lobe interhemispheric functional connectivity in adolescents with internet gaming disorder: a primary study using resting-state FMRI. PLoS ONE. (2015) 10:e0118733. 10.1371/journal.pone.0118733 25738502 PMC4349655

[16] Kim H Kim YK Gwak AR Lim JA Lee JY Jung HY . Resting-state regional homogeneity as a biological marker for patients with Internet gaming disorder: a comparison with patients with alcohol use disorder and healthy controls. Progress Neuro Psychopharmacol Biol Psychiatry. (2015) 60:104–11. 10.1016/j.pnpbp.2015.02.004 25689820

[17] Radua J Mataix-Cols D. Voxel-wise meta-analysis of grey matter changes in obsessive-compulsive disorder. Br J Psychiatry. (2009) 195:393–402. 10.1192/bjp.bp.108.055046 19880927

[18] Radua J Mataix-Cols D. Meta-analytic methods for neuroimaging data explained. Biol Mood Anxiety Disord. (2012) 2:6. 10.1186/2045-5380-2-6 22737993 PMC3384225

[19] Dugré JR Radua J Carignan-Allard M Dumais A Rubia K Potvin S. Neurofunctional abnormalities in antisocial spectrum: a meta-analysis of fMRI studies on Five distinct neurocognitive research domains. Neurosci Biobehav Rev. (2020) 119:168–83. 10.1016/j.neubiorev.2020.09.013 32956690

[20] Li H Chen Z Gong Q Jia Z. Voxel-wise meta-analysis of task-related brain activation abnormalities in major depressive disorder with suicide behavior. Brain Imaging Behav. (2020) 14:1298–308. 10.1007/s11682-019-00045-3 30790165

[21] Meng YJ Deng W Wang HY Guo WJ Li T Lam C . Reward pathway dysfunction in gambling disorder: a meta-analysis of functional magnetic resonance imaging studies. Behav Brain Res. (2014) 275:243–51. 10.1016/j.bbr.2014.08.057 25205368

[22] Meng YJ Deng W Wang HY Guo WJ Li T. The prefrontal dysfunction in individuals with Internet gaming disorder: a meta-analysis of functional magnetic resonance imaging studies. Addict Biol. (2015) 20:799–808. 10.1111/adb.12154 24889021

[23] Yao YW Liu L Ma SS Shi XH Zhou N Zhang JT . Functional and structural neural alterations in Internet gaming disorder: a systematic review and meta-analysis. Neurosci Biobehav Rev. (2017) 83:313–24. 10.1016/j.neubiorev.2017.10.029 29102686

[24] Zheng H Hu YB Wang ZL Wang M Du XX Dong GH. Meta-analyses of the functional neural alterations in subjects with Internet gaming disorder: similarities and differences across different paradigms. Progress Neuro Psychopharmacol Biol Psychiatry. (2019) 94:109656. 10.1016/j.pnpbp.2019.109656 31145927

[25] Moher D Liberati A Tetzlaff J Altman DG. Preferred reporting items for systematic reviews and meta-analyses: the PRISMA statement. BMJ. (2009) 339:b2535. 10.1136/bmj.b2535 19622551 PMC2714657

[26] Stang A. Critical evaluation of the Newcastle-Ottawa scale for the assessment of the quality of nonrandomized studies in meta-analyses. Eur J Epidemiol. (2010) 25:603–5. 10.1007/s10654-010-9491-z 20652370

[27] Yuhara H Steinmaus C Cohen SE Corley DA Tei Y Buffler PA. Is diabetes mellitus an independent risk factor for colon cancer and rectal cancer? Am J Gastroenterol. (2011) 106:1911–21. 10.1038/ajg.2011.301 21912438 PMC3741453

[28] Radua J Via E Catani M Mataix-Cols D. Voxel-based meta-analysis of regional white-matter volume differences in autism spectrum disorder versus healthy controls. Psychol Med. (2011) 41:1539–50. 10.1017/s0033291710002187 21078227

[29] Radua J Mataix-Cols D Phillips ML El-Hage W Kronhaus DM Cardoner N . A new meta-analytic method for neuroimaging studies that combines reported peak coordinates and statistical parametric maps. Eur Psychiatry. (2012) 27:605–11. 10.1016/j.eurpsy.2011.04.001 21658917

[30] Li Q Zhao Y Chen Z Long J Dai J Huang X . Meta-analysis of cortical thickness abnormalities in medication-free patients with major depressive disorder. Neuropsychopharmacology. (2020) 45:703–12. 10.1038/s41386-019-0563-9 31694045 PMC7021694

[31] Pezzoli S Emsell L Yip SW Dima D Giannakopoulos P Zarei M . Meta-analysis of regional white matter volume in bipolar disorder with replication in an independent sample using coordinates, T-maps, and individual MRI data. Neurosci Biobehav Rev. (2018) 84:162–70. 10.1016/j.neubiorev.2017.11.005 29162519 PMC5771263

[32] Du X Yang YX Gao PH Qi X Du G Zhang Y . Compensatory increase of functional connectivity density in adolescents with internet gaming disorder. Brain Imaging Behav. (2017) 11:1901–9. 10.1007/s11682-016-9655-x 27975158

[33] Feng Q Chen X Sun JH Zhou Y Sun Y Ding W . Voxel-level comparison of arterial spin-labeled perfusion magnetic resonance imaging in adolescents with internet gaming addiction. Behav Brain Funct. (2013) 9:33. 10.1186/1744-9081-9-33 23937918 PMC3751515

[34] Han X Wang Y Jiang WQ Bao X Sun Y Ding W . Resting-state activity of prefrontal-striatal circuits in internet gaming disorder: changes with cognitive behavior therapy and predictors of treatment response. Front Psychiatry. (2018) 9:341. 10.3389/fpsyt.2018.00341 30123144 PMC6085723

[35] Liu L Potenza MN Lacadie CM Zhang JT Yip SW Xia CC . Altered intrinsic connectivity distribution in internet gaming disorder and its associations with psychotherapy treatment outcomes. Addict Biol. (2020) 26:e12917. 10.1111/adb.12917 32415913

[36] Wang L Shen H Lei Y Zeng LL Cao F Su L . Altered default mode, fronto-parietal and salience networks in adolescents with Internet addiction. Addict Behav. (2017) 70:1–6. 10.1016/j.addbeh.2017.01.021 28160660

[37] Wang Y Qin Y Li H Yao D Sun B Li Z . Abnormal functional connectivity in cognitive control network, default mode network, and visual attention network in internet addiction: a resting-state fMRI study. Front Neurol. (2019) 10:1006. 10.3389/fneur.2019.01006 31620077 PMC6759465

[38] Beard KW Wolf EM. Modification in the proposed diagnostic criteria for Internet addiction. Cyberpsychol Behav. (2001) 4:377–83. 10.1089/109493101300210286 11710263

[39] Lee J Lee D Namkoong K Jung YC. Aberrant posterior superior temporal sulcus functional connectivity and executive dysfunction in adolescents with internet gaming disorder. J Behav Addict. (2020) 9:589–97. 10.1556/2006.2020.00060 32918802 PMC8943665

[40] Jackson RL Bajada CJ Rice GE Cloutman LL Lambon Ralph MA. An emergent functional parcellation of the temporal cortex. Neuroimage. (2017) 170:385–99. 10.1016/j.neuroimage.2017.04.024 28419851

[41] Achiron A Chapman J Tal S Bercovich E Gil H Achiron A. Superior temporal gyrus thickness correlates with cognitive performance in multiple sclerosis. Brain Struct Funct. (2013) 218:943–50. 10.1007/s00429-012-0440-3 22790785

[42] Chen B. Abnormal cortical regions and subsystems in whole brain functional connectivity of mild cognitive impairment and Alzheimer's disease: a preliminary study. Aging Clin Exp Res. (2021) 33:367–81. 10.1007/s40520-020-01539-7 32277436

[43] Cai C Huang C Yang C Zhang X Peng Y Zhao W . Altered patterns of phase position connectivity in default mode subnetwork of subjective cognitive decline and amnestic mild cognitive impairment. Front Neurosci. (2020) 14:185. 10.3389/fnins.2020.00185 32265623 PMC7099636

[44] Li W Qin W Liu H Fan L Wang J Jiang T . Subregions of the human superior frontal gyrus and their connections. Neuroimage. (2013) 78:46–58. 10.1016/j.neuroimage.2013.04.011 23587692

[45] Buckner RL Kelley WM Petersen SE. Frontal cortex contributes to human memory formation. Nat Neurosci. (1999) 2:311–4. 10.1038/7221 10204536

[46] Tamminga CA Buchsbaum MS. Frontal cortex function. Am J Psychiatry. (2004) 161:2178. 10.1176/appi.ajp.161.12.2178 15569885

[47] Wang CG Zhang ZY Che LP Wu YY Qian HY Guo XY. The gray matter volume in superior frontal gyrus mediates the impact of reflection on emotion in Internet gaming addicts. Psychiatry Res Neuroimaging. (2021) 310:111269. 10.1016/j.pscychresns.2021.111269 33657478

[48] Ding WN Sun JH Sun YW Chen X Zhou Y Zhuang ZG . Trait impulsivity and impaired prefrontal impulse inhibition function in adolescents with internet gaming addiction revealed by a Go/No-Go fMRI study. Behav Brain Funct. (2014) 10:20. 10.1186/1744-9081-10-20 24885073 PMC4050412

[49] Wang YF Wu LD Zhou HL Lin X Zhang Y Du X . Impaired executive control and reward circuit in Internet gaming addicts under a delay discounting task: independent component analysis. Eur Arch Psychiatry Clin Neurosci. (2017) 267:245–55. 10.1007/s00406-016-0721-6 27506757

[50] Li Y Li G Liu L Wu H. Correlations between mobile phone addiction and anxiety, depression, impulsivity, and poor sleep quality among college students: a systematic review and meta-analysis. J Behav Addict. (2020) 9:551–71. 10.1556/2006.2020.00057 32903205 PMC8943681

[51] Cheng HW Liu J. Alterations in amygdala connectivity in internet addiction disorder. Sci Rep. (2020) 10:2370. 10.1038/s41598-020-59195-w 32047251 PMC7012850

[52] Ko CH Hsieh TJ Wang PW Lin WC Yen CF Chen CS . Altered gray matter density and disrupted functional connectivity of the amygdala in adults with Internet gaming disorder. Progress Neuro Psychopharmacol Biol Psychiatry. (2015) 57:185–92. 10.1016/j.pnpbp.2014.11.003 25448779

[53] Dean SF Fede SJ Diazgranados N Momenan R. Addiction neurocircuitry and negative affect: a role for neuroticism in understanding amygdala connectivity and alcohol use disorder. Neurosci Lett. (2020) 722:134773. 10.1016/j.neulet.2020.134773 32045624

[54] Kallupi M Wee S Edwards S Whitfield TW Jr Oleata CS Luu G . Kappa opioid receptor-mediated dysregulation of gamma-aminobutyric acidergic transmission in the central amygdala in cocaine addiction. Biol Psychiatry. (2013) 74:520–8. 10.1016/j.biopsych.2013.04.028 23751206 PMC3773286

[55] Sharp BM. Basolateral amygdala and stress-induced hyperexcitability affect motivated behaviors and addiction. Transl Psychiatry. (2017) 7:e1194. 10.1038/tp.2017.161 28786979 PMC5611728

[56] Janiri D Moser DA Doucet GE Luber MJ Rasgon A Lee WH . Shared neural phenotypes for mood and anxiety disorders: a meta-analysis of 226 task-related functional imaging studies. JAMA Psychiatry. (2020) 77:172–9. 10.1001/jamapsychiatry.2019.3351 31664439 PMC6822098

[57] Wang C Wang Y Lau WKW Wei X Feng X Zhang C . Anomalous static and dynamic functional connectivity of amygdala subregions in individuals with high trait anxiety. Depress Anxiety. (2021) 38:860–73. 10.1002/da.23195 34254391

[58] Baxter MG Murray EA. The amygdala and reward. Nat Rev Neurosci. (2002) 3:563–73. 10.1038/nrn875 12094212

[59] Kawasaki K Annicchiarico I Glueck AC Morón I Papini MR. Reward loss and the basolateral amygdala: a function in reward comparisons. Behav Brain Res. (2017) 331:205–13. 10.1016/j.bbr.2017.05.036 28511980

[60] Stuber GD Sparta DR Stamatakis AM van Leeuwen WA Hardjoprajitno JE Cho S . Excitatory transmission from the amygdala to nucleus accumbens facilitates reward seeking. Nature. (2011) 475:377–80. 10.1038/nature10194 21716290 PMC3775282

[61] Seeley WW. The salience network: a neural system for perceiving and responding to homeostatic demands. J Neurosci. (2019) 39:9878–82. 10.1523/jneurosci.1138-17.2019 31676604 PMC6978945

[62] Dunlop K Hanlon CA Downar J. Noninvasive brain stimulation treatments for addiction and major depression. Ann N Y Acad Sci. (2017) 1394:31–54. 10.1111/nyas.12985 26849183 PMC5434820

[63] Grodin EN Cortes CR Spagnolo PA Momenan R. Structural deficits in salience network regions are associated with increased impulsivity and compulsivity in alcohol dependence. Drug Alcohol Depend. (2017) 179:100–8. 10.1016/j.drugalcdep.2017.06.014 28763777 PMC11034794

[64] Taylor KS Kucyi A Millar PJ Murai H Kimmerly DS Morris BL . Association between resting-state brain functional connectivity and muscle sympathetic burst incidence. J Neurophysiol. (2016) 115:662–73. 10.1152/jn.00675.2015 26538607 PMC4752303

[65] Wang S Liu J Tian L Chen L Wang J Tang Q . Increased insular cortical thickness associated with symptom severity in male youths with internet gaming disorder: a surface-based morphometric study. Front Psychiatry. (2018) 9:99. 10.3389/fpsyt.2018.00099 29666588 PMC5891580

[66] Lee D Namkoong K Lee J Jung YC. Dorsal striatal functional connectivity changes in Internet gaming disorder: a longitudinal magnetic resonance imaging study. Addict Biol. (2021) 26:e12868. 10.1111/adb.12868 31886611

[67] Zhou Y Lin FC Du YS Qin LD Zhao ZM Xu JR . Gray matter abnormalities in Internet addiction: a voxel-based morphometry study. Eur J Radiol. (2011) 79:92–5. 10.1016/j.ejrad.2009.10.025 19926237

[68] He Q Turel O Bechara A. Brain anatomy alterations associated with Social Networking Site (SNS) addiction. Sci Rep. (2017) 7:45064. 10.1038/srep45064 28332625 PMC5362930

[69] Starcke K Antons S Trotzke P Brand M. Cue-reactivity in behavioral addictions: a meta-analysis and methodological considerations. J Behav Addict. (2018) 7:227–38. 10.1556/2006.7.2018.39 29788752 PMC6174580

[70] Dong G Liu X Zheng H Du X Potenza MN. Brain response features during forced break could predict subsequent recovery in internet gaming disorder: a longitudinal study. J Psychiatr Res. (2019) 113:17–26. 10.1016/j.jpsychires.2019.03.003 30878788

[71] Turel O He QH Wei L Bechara A. The role of the insula in internet gaming disorder. Addict Biol. (2021) 26:e12894. 10.1111/adb.12894 32147952

[72] Tolomeo S Yu R. Brain network dysfunctions in addiction: a meta-analysis of resting-state functional connectivity. Transl Psychiatry. (2022) 12:41. 10.1038/s41398-022-01792-6 35091540 PMC8799706

[73] Thomson H Labuschagne I Greenwood LM Robinson E Sehl H Suo C . Is resting-state functional connectivity altered in regular cannabis users? A systematic review of the literature. Psychopharmacology. (2021). 10.1007/s00213-021-05938-0. [Epub ahead of print]. 34415377

[74] Lei W Liu K Chen G Tolomeo S Liu C Peng Z . Blunted reward prediction error signals in internet gaming disorder. Psychol Med. (2020) 1–10. 10.1017/S003329172000402X. [Epub ahead of print]. 33143778

[75] Wang M Dong H Zheng H Du X Dong GH. Inhibitory neuromodulation of the putamen to the prefrontal cortex in Internet gaming disorder: how addiction impairs executive control. J Behav Addict. (2020) 9:312–24. 10.1556/2006.2020.00029 32663381 PMC8939425

[76] Zang Y Jiang T Lu Y He Y Tian L. Regional homogeneity approach to fMRI data analysis. Neuroimage. (2004) 22:394–400. 10.1016/j.neuroimage.2003.12.030 15110032

[77] Zou QH Zhu CZ Yang Y Zuo XN Long XY Cao QJ . An improved approach to detection of amplitude of low-frequency fluctuation (ALFF) for resting-state fMRI: fractional ALFF. J Neurosci Methods. (2008) 172:137–41. 10.1016/j.jneumeth.2008.04.012 18501969 PMC3902859

[78] Borogovac A Asllani I. Arterial Spin Labeling (ASL) fMRI: advantages, theoretical constrains, and experimental challenges in neurosciences. Int J Biomed Imaging. (2012) 2012:818456. 10.1155/2012/818456 22966219 PMC3432878

[79] Lau WK Leung MK Lee TM Law AC. Resting-state abnormalities in amnestic mild cognitive impairment: a meta-analysis. Transl Psychiatry. (2016) 6:e790. 10.1038/tp.2016.55 27115121 PMC4872413

[80] Xia W Chen YC Ma J. Resting-state brain anomalies in type 2 diabetes: a meta-analysis. Front Aging Neurosci. (2017) 9:14. 10.3389/fnagi.2017.00014 28197096 PMC5281680

