# Supplementary material for: Functional Neural Alterations in Pathological Internet Use: A Meta-Analysis of Neuroimaging Studies
Source: Front Neurol. 2022 Apr 18;13:841514. doi: 10.3389/fneur.2022.841514 (PMC9062178; doi:10.3389/fneur.2022.841514)
Supplement: Supplementary file 1 [file Table_1.docx]

**Table S1. Quality ratings for the 8 case-control studies included on the basis of Newcastle-Ottawa scale**

| **Study name** | **Selection** | | | | **Comparability** | **Outcome** | | | **Total score** |
| --- | --- | --- | --- | --- | --- | --- | --- | --- | --- |
|  | **(1)** | **(2)** | **(3)** | **(4)** | **(5)** | **(6)** | **(7)** | **(8)** |  |
| Xin Du (2016) |  | * |  | * | ** |  | * |  | 5 |
| Qi Feng (2013) | * | * |  | * | ** |  | * |  | 6 |
| Heejung Kim (2015) | * | * | * | * | ** |  | * |  | 7 |
| Jun Liu (2010) |  | * |  | * | ** |  | * |  | 5 |
| Yawen Sun (2018) |  | * | * | * | ** |  | * |  | 6 |
| Yao Wang (2015) | * | * |  | * | ** |  | * |  | 6 |
| Lubin Wang (2017) | * | * | * | * | ** |  | * | * | 8 |
| Yang Wang (2019) |  | * | * | * | ** |  | * |  | 6 |
| Xu Han (2018) |  | * | * | * | ** |  | * |  | 6 |
| Lu Liu (2020) |  | * | * | * | ** |  | * |  | 6 |
| mean | | | | | | | | | 6.1 |

**Note:** (1) Is the case definition adequate? (2) Representativeness of the cases; (3) Selection of Controls; (4) Definition of Controls; (5) Comparability of cases and controls on the basis of the design or analysis; (6) Ascertainment of exposure; (7) Same method of ascertainment for cases and controls; (8) Non-Response rate
