# Supplementary material for: Functional Neural Alterations in Pathological Internet Use: A Meta-Analysis of Neuroimaging Studies
Source: Front Neurol. 2022 Apr 18;13:841514. doi: 10.3389/fneur.2022.841514 (PMC9062178; doi:10.3389/fneur.2022.841514)
Supplement: Supplementary file 2 [file Table_2.docx]

**Table S2. Quality ratings for the 2 cohort studies included on the basis of Newcastle-Ottawa scale**

| **Study name** | **Selection** | | | | **Comparability** | **Outcome** | | | **Total score** |
| --- | --- | --- | --- | --- | --- | --- | --- | --- | --- |
|  | **(1)** | **(2)** | **(3)** | **(4)** | **(5)** | **(6)** | **(7)** | **(8)** |  |
| Xu Han (2018) |  |  | * | * | ** | * |  |  | 5 |
| Lu Liu (2020) |  |  | * |  | ** | * |  | * | 5 |
| mean | | | | | | | | | 5 |

**Note:** (1) Representativeness of the exposed cohort; (2) Selection of the non-exposed cohort; (3) Ascertainment of exposure; (4) Demonstration that outcome of interest was not present at start of study; (5) Comparability of cohorts on the basis of the design or analysis; (6) Assessment of outcome; (7) Was follow-up long enough for outcomes to occur; (8) Adequacy of follow up of cohorts
